# Evaluation of antimicrobial and cytotoxic effects of *Echinacea* and *Arctium* extracts and *Zataria* essential oil

**DOI:** 10.1186/s13568-022-01417-7

**Published:** 2022-06-15

**Authors:** Mohsen Yazdanian, Pouya Rostamzadeh, Mostafa Alam, Kamyar Abbasi, Elahe Tahmasebi, Hamid Tebyaniyan, Reza Ranjbar, Alexander Seifalian, Mehrdad Moosazadeh Moghaddam, Majid Balaei Kahnamoei

**Affiliations:** 1 Research Center for Prevention of Oral and Dental Diseases, Baqiyatallah University of Medical Sciences, Tehran, Iran; 2 School of Dentistry, Baqiyatallah University of Medical Sciences, Tehran, Iran; 3 Scientific Research Center (DSSRC), Tehran University of Medical Sciences, Tehran, Iran; 4 Department of Oral and Maxillofacial Surgery, School of Dentistry, Shahid Beheshti University of Medical Sciences, Tehran, Iran; 5 Department of Prosthodontics, School of Dentistry, Shahid Beheshti University of Medical Sciences, Tehran, Iran; 6 Science and Research Branch, Islamic Azad University, Tehran, Iran; 7 Nanotechnology and Regenerative Medicine, Commercialization Centre (NanoRegMed Ltd), The London Bioscience Innovation Centre, London, UK; 8 Applied Biotechnology Research Center, Baqiyatallah University of Medical Sciences, Tehran, Iran; 9 Department of Pharmacognosy, Faculty of Pharmacy, Tehran University of Medical Sciences, Tehran, Iran

**Keywords:** Antimicrobial, Cytotoxicity, *Zataria multiflora*, *Echinacea purpurea*, *Arctium lappa*

## Abstract

Dental caries and oral infections have become a widespread issue in the modern world. This study aimed to investigate the antibacterial, antifungal, and cytotoxicity characteristics of the extracts of *Echinacea purpura*, *Arctium lappa*, and the essential oil of *Zataria multiflora* as a potential herbal mouthwash. The essential oil of *Z. multiflora* leaves and the extracts of *E. purpurea and A. lappa* roots were prepared. The characterization was carried out by GC-MS and also, total phenol and flavonoid were assed for all three samples. The antimicrobial and anti-biofilm effects were evaluated against *Streptococcus mutans, Streptococcus mitis, Streptococcus salivarius, Lactobacillus acidophilus, Escherichia coli, Staphylococcus aureus,* and *Candida albicans.* The cytotoxic effect of the samples was evaluated on HEK 293 and HDFa cells by MTT test. Thymol and carvacrol contents in EO of *Z. multiflora* were measured at 31% and 42.2%, respectively. *A. lappa* had the lowest total phenolic and flavonoid value among the samples. On the other hand, the total phenolic content of *Z. multiflora* and the total flavonoid content of *E. purpurea* were the highest. The MIC values of *Zataria*, *Arctium*, and *Echinacea* against *S. mutans* were 0.011% v/v, 187.5 mg/ml, and 93.75 mg/ml, while MBC were 0.011% v/v, 375 mg/ml, and 187.5 mg/ml, respectively. The formulation showed bactericidal activity against *S. mutans* in the concentration of 5.86 mg/ml for Echinacea and Burdock extracts and 0.08 µl/ml for EO of *Zataria.* The formulation significantly affected microbial biofilm formation and induced biofilm degradation. The cell viability percentages were higher than 50% during 24 and 48 h. The formulation had a significant antimicrobial effect on cariogenic bacteria and *C. albicans*, with the lowest cytotoxic effects. Therefore, this formulation can be an appropriate candidate for mouthwash.

## Introduction

Dental caries is a disease in which an ecological shift within the dental biofilm environment, fueled by frequent access to fermentable dietary carbohydrates, causes a shift from a balanced population of low cariogenic microorganisms to a microbiological population of high cariogenicity (more aciduric and acidogenic), as well as an increase in the production of organic acids. This causes net mineral loss in the dental hard tissue, resulting in a carious lesion (Innes et al. 2016). Poor oral hygiene, dental caries, and periodontal infections have been linked to various illnesses, including gastrointestinal and cardiovascular diseases, as well as preterm labor and low-birthweight babies (Romero et al. 2002; Beck et al. 2005; Jia et al. 2009). Moreover, the costs of inflammatory mouth diseases and tooth decay to a country's health system are enormous. According to one report, the total direct and indirect costs (absenteeism and reduced productivity) of these diseases are estimated at $ 422 billion per year worldwide, underscoring the importance of oral health (Listl et al. 2015). Therefore, the best way to reduce these direct and indirect costs is to prevent caries and gingivitis. According to the etiology of dental caries, the formation of dental plaque is the first step in developing gingivitis and dental caries. Dental plaque is considered a dense and non-calcified microbial accumulation that adheres to tooth surfaces that are not washed away by saliva flow (Rosan et al. 2000). Therefore, preventing plaque formation and removing plaque formed on dental surfaces is the best way to promote oral health and prevent tooth decay. Mechanical and chemical methods are recommended to remove formed plaque from the teeth. Brushing as a mechanical method of removing plaque is considered the most accessible method of preventing dental caries (Wootton 2005; Jakubovics et al. 2021). Due to the inadequacy of mechanical methods in the comprehensive removal of plaques, chemical methods are also used to complete the mechanical removal. Mouthwashes are the most common method in the chemical control of plaque (Madhavi et al. 2020).

Mouthwashes are classified as either chemical (synthetic) or herbal. Chemical mouthwashes are available in a variety of forms. Chlorhexidine mouthwash as a synthetic product is widely used due to its wide range of antimicrobial effects and low cost. Chlorhexidine works against bacteria by disrupting their cell membranes (Azgomi et al. 2016). Despite chlorhexidine's antimicrobial and anti-inflammatory properties, its numerous side effects, such as tooth staining, unpleasant taste in the mouth, mouth irritation, gingivitis, and side effects from potential swallowing, have limited its usage (Kumar 2017). Therefore, it is necessary to do more studies to find more suitable alternatives with better therapeutic effects and fewer side effects. According to a study conducted by the World Health Organization in 2013, more than 80% of the world's population relies on traditional medicines for their primary health needs (Qi 2013). Because of the long tradition of people's interaction with nature, medicinal plants have played a significant role in treating diseases in Iran. Iran has a diverse flora and a wealth of information about its natural medicinal plants. Native plants with antimicrobial properties are used in rural villages in Iran, but there is no scientific evidence to prove them. As a result, screening and research into these herbs could lead to the discovery of new antimicrobial compounds (Amiri et al. 2021). In recent years, the use of herbal medicines has increased significantly in many areas of health and wellness, but in oral health, the use of herbs is limited. Despite various laboratory and clinical studies on different medicinal plants, there are limited commercial products in this field (Cruz Martinez et al. 2017; Anushya et al. 2020). The most well-known herbal mouthwash is Listerine, which contains four essential oils menthol, thymol, eucalyptol, methyl salicylate, and 27% ethanol (Vlachojannis et al. 2015).

*Zataria multiflora* (Avishan-e-Shirazi) from the *Lamiaceae* family has been known as a plant in Iranian food and medicine culture for a long time and has been used more to relieve gastrointestinal disorders. However, numerous articles have been published on its antimicrobial and anti-inflammatory. Antioxidant properties, immune system stimulant, and antispasmodic, and good results have been reported. Also the antibacterial effect of essential oils of this plant on pathogenic caries has been shown in oral and dental health (Hashemi et al. 2017; Khazdair et al. 2018; Saeidi et al. 2019). *Zataria multiflora* is the only native thyme species in Iran and grows in Isfahan, Lorestan, Khuzestan, Fars, Bushehr, Kerman, Hormozgan, Baluchistan, Khorasan, and Yazd. In contemporary medicine, the root of *Arctium* (Baba Adam, Burdock) from the *Asteraceae* family is used as a diuretic and anti-inflammatory agent. In traditional medicine, the first-year leaves of the plant are widely used as anti-inflammatory, anti-cancer, diuretic, diaphoretic, and choleretic agents (Ahangarpour et al. 2017; Nascimento et al. 2019; Zhang et al. 2019). There have also been studies on diabetes that have shown that it lowers blood sugar. It has also been used externally for wound healing, regenerative agents, and tissue regeneration. Researchers have proven the antibacterial effect of this plant (Ahangarpour et al. 2017; Nascimento et al. 2019; Zhang et al. 2019). These plants are cultivated in Tehran, Alborz, Khorasan, Kerman, Rudbar, Tafresh, Europe, and North America. Numerous clinical studies have been performed on *Echinacea* from the *Asteraceae* family to evaluate the preventive or therapeutic effect on infections and the modulating effect on the immune system. These studies have claimed that plant-based products have a significant effect on stimulating the immune system. *Echinacea* has been shown to affect the severity and duration of gingivitis and dental plaque. This herbal medicine generally has no side effects, and even swallowing could not cause the related problems (Dronyk et al. 2017; Billah et al. 2019; Izadi et al. 2020). While it is not native to Iran, it has been introduced to native Iranian plants in recent years. This plant's antimicrobial properties have recently been recognized and attributed to the presence of phenolic compounds (Fathi et al. 2021). In this study, the cytotoxicity and antimicrobial effects of the essential oil, herbal extracts, and final formulation as mouthwash (*Zataria multiflora, Echinacea purpura,* and *Arctium lappa*) were evaluated on fibroblast cells/HEK 293 and *Streptococcus mutans, Streptococcus mitis, Streptococcus salivarius, Lactobacillus acidophilus, Escherichia coli, Staphylococcus aureus,* and *Candida albicans*, respectively.

## Materials and methods

### Materials

*Streptococcus mutans* (ATCC: 35,668)*, Streptococcus mitis* (ATCC: 6249)*, Streptococcus salivarius* (ATCC: 13,419)*, Lactobacillus acidophilus* (ATCC:314)*, Candida albicans* (89–1093), *Staphylococcus aureus* (ATCC: 29,213)*, and Escherichia coli* (ATCC: 25,922), Human embryonic kidney cells (HEK 293, NCBI: C497) and Human Dermal fibroblast cells (HDFa) (HFSF-PI 3, NCBI: C167) were gifted from Baqiyatallah University of Medical Sciences, Tehran, Iran. Brain Heart Infusion (BHI) broth and agar and Chrystal violet were provided from Merck (Darmstadt, Germany). MTT Kit was obtained from Bioidea (Iran). Dimethyl sulfoxide (DMSO) was obtained from Sigma‐Aldrich. Dulbecco's modified Eagle's medium (DMEM), Fetal bovine serum (FBS), phosphate‐buffered saline (PBS), trypsin, anti‐streptomycin, and beta-glycerol were purchased from Gibco (New York, USA).

## Plant sampling and extraction

### *Zataria multiflora*

**Fig. 1:**
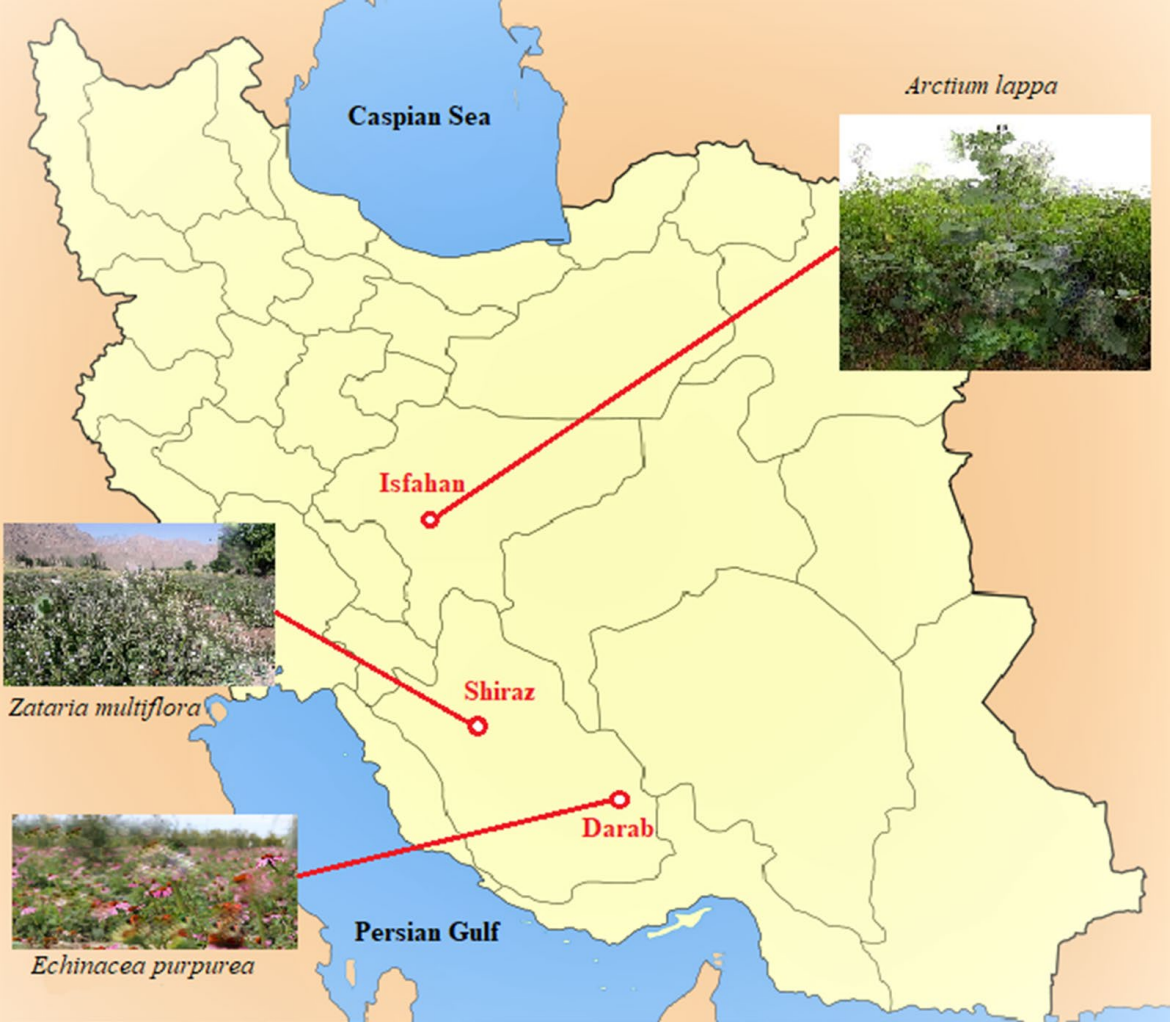
Locations of farms for sampling

The aerial parts of *Zataria multiflora* were collected from rural districts of Fars province (29°33′17″ N, 52°41′02E”) near Shiraz (Fig. 1). A botanist identified the plant at the Tehran University of Medical Sciences, Tehran, Iran. One kilogram of aerial parts was dried in the shade and pulverized. An all-glass Clevenger type apparatus performed Hydro-distillation of the plant for about 3 h. The obtained essential oil was dehydrated by anhydrous sodium sulfate and kept in a closed container away from light in a refrigerator at 4 °C in up to use (Sadeghi et al. 2015). The essential oil color was light yellow, and it had a strong, penetrating thyme odor.

### Echinacea purpura

The root of *E. purpura* was received from Darab, Fars, Iran (28°45′26″ N, 54°31′07″ E) (Fig. 1). Chopped plant material (300 mg) was immersed in 1.2 L of 70% EtOH solution for 3 days, and during this time, the extract was stirred twice. After 3 days, it was filtered, and for the remaining plant, this process was repeated two more times. Finally, the ethanol was removed using a rotary evaporator [BUCHI Rotavapor R-114, Germany], and the extracts were separated by lyophilization using a freeze-drier HETOSICC, Heto Lab Equipment, Denmark. A total of 29 g of dry brown extract was obtained.

### *Arctium lappa*

Burdock root was received from Isfahan, Iran (29°33′17″ N, 52°41′02″) (Fig. 1) and approved by the botanist of the Faculty of Pharmacy of Tehran University of Medical Sciences. The dried roots were cut into pieces, and 500 g was added into 2 L of 40% EtOH and kept at room temperature for 3 days. During this time, the mixture was stirred twice. The mixture was filtered, and for the remaining plant, these steps were repeated two more times. Finally, a rotary evaporator was used to remove the solvent, and the water was removed through the freeze-drier to have 25 g lyophilized extract.

## Phytochemical screening

### Measurement of the total amount of phenolic compounds

The measurement was performed according to Wolfe et al. (2003) method with some changes. 2 ml of Folin-Ciocalteu diluted 1: 10 with water was added to 0.5 ml of each extract and then sodium carbonate (75 g/L, 2 mL). The mixture was centrifuged for 15 s and left at 30 °C for 30 min to complete staining. The absorption associated with the 765 nm wavelength was measured. Gallic acid was used to calculate the standard chromium (R^2^ = 0.999, 0.05–0.8 mM: y = 1.683x + 0.044). Results were reported in mg equivalent of Gallic acid (GAE) per ml of extract solution (Wolfe et al. 2003).

### Measurement of the total amount of flavonoid compounds

The measurement was performed according to Jia et al. (1999) method with some changes. 5 ml of each extract was mixed with deionized water (2 ml), and then sodium nitrite solution (5%, 0.15 mL) was added. After 6 min, AlCL3 solution (10%, 0.15 ml) was added and left for 6 min longer. Then, sodium hydroxide (4%, 2 ml) and deionized water were added to make a final volume of 5 ml. Finally, the resulting solution was left for 15 min, and the pink color intensity was calculated at a wavelength of 510 nm. Catechin was used to calculate the standard chromium (0.0156–1.0182 mM; y = 0.98766x—0.0008; R^2^ = 0.999) and the results were reported in milligrams equivalent to catechin per milliliter of extract solution (Zhishen et al. 1999).

### GC-MS detection of thymol and carvacrol in the essential oil

For GC-MS analysis, an electron ionization system was used with a 70 eV ionization energy. As a carrier gas, helium gas was used at a constant flow rate of 1 ml.min^−1^. The injector and mass transfer line temperatures were set to 250 °C and 300 °C, respectively. The oven temperature was programmed to rise from 50 to 200 °C at the constant pace of 8 °C min^−1^, then kept isothermally for 20 min before being lifted to 300 °C at 10 °C min^−1^. In the splitless mode, diluted samples (1/100 v/v, in methanol) of 0.2 µl were manually injected. GC retention time on the VF-5 capillary column and algorithm matching of mass spectra with standards were used to identify essential oil compounds. Wiley's library analysis and the ISIRI No 5192 determined the form of components entering mass spectrometry (Saei-Dehkordi et al. 2010).

## Cytotoxicity study

### Cell culture

Human embryonic kidney cells (HEK 293) were grown in Dulbecco's modified eagle medium (DMEM) containing 10% fetal bovine serum (FBS), streptomycin sulfate (10 mg/ml), and penicillin G sodium (10 units/ml) at 37 °C in a humidified incubator with 5% CO2 and 90% humidity. The culture media was replaced every 48 h (80 percent). Next, cells were used from the second passage.

### Cell viability test

The cytotoxic effects of the extracts, the essential oil, and the formulation on Human Dermal Fibroblast cells (HDFa) and Human Embryonic Kidney cells (HEK 293) (Pasteur Institute, Tehran, Iran) were measured by 3-(4, 5-dimethylthiazol-2-yl)-2, 5-diphenyltetrazolium bromide (MTT) assay method (ISO 10993-5). Cells were incubated for 24 h before adding to 96 plates. Cells cultured without extracts were used as a control. After 24 and 48 h, the solution was replaced with 5 mg/mL MTT. Then, the MTT solution was removed, and after 3 h, 0.1 mL isopropanol was added to dissolve the Formazan product. Their absorption was monitored at 570 nm on a microplate reader. The findings were presented as percentages (control value = 100%). These tests were performed three times. The percentage of cell viability was measured using the following equation:

$$\mathrm{The\,percentage\,of\,Cell\,viability}=\frac{\mathrm{Samples\,}(\mathrm{OD})}{\mathrm{Control\,}(\mathrm{OD}) }\times 100$$

## Antimicrobial tests

### Preparation of final sample concentration for the antimicrobial tests

To obtain the solution, 1.5% (v/v) of *Zataria multiflora* essential oil was prepared with distilled water. Also, a 1.5 g/ml concentration was prepared from lyophilized extracts of Echinacea and Arctium.

### Bacterial strain

The bacterial strains used in this experiment were *Streptococcus mutans, Streptococcus mitis, Streptococcus salivarius, Lactobacil acidophilus, Staphylococcus aureus, and Escherichia coli*. The cultures were stored at −80 °C in brain heart infusion (BHI) containing 20% glycerol (v/v). The concentration of the strains in the study was adjusted to a final concentration of 0.5 McFarland (1.5 × 10^8^ CFU/mL) before the experiment. The stock culture of *C. albicans* was inoculated initially into 30 mL of sterilized Yeast Peptone Dextrose (YPD) broth to form the *C. albicans* suspension. The concentration of *C. albicans* in the study was adjusted to a final concentration of 0.5 McFarland (1.5 × 10^6^ CFU/mL) before the experiment.

### Determination of minimum inhibitory concentration (MIC), minimum bactericidal concentration (MBC), and minimal fungicidal concentration (MFC) tests

MIC and MBC were measured using the broth microdilution method. Serial doubling dilutions of the extract suspensions were prepared in a 96-well microtiter plate. The final concentration of each strain was adjusted to 10^5^–10^6^ CFU/ml. All microtiter plates were incubated for 24 h at 37 °C. After incubation, wells were examined for the growth of microorganisms, and the MIC was determined. MIC was defined as the lowest concentration of the extracts tested, showing restricted growth at a lower level than 0.05 at 660 nm (no visible growth). To determine MBC, an aliquot (50 ml) of each incubated tube containing extract suspension at concentrations higher than the MIC was sub-cultured on BHI agar supplemented with 5% defibrinated horse blood using a Spiral Plater (Whitley Automatic Spiral Plater). MBC and MFC were defined as the lowest concentration of essential oil or dry extracts in which incubating microorganisms are entirely killed. Each experiment was repeated three times. Chlorhexidine digluconate 0.2% (v/v) was used as a positive control for determining MIC and MBC. After obtaining MIC and MBC extracts, the required concentration of each extract in the formulation was obtained. MIC and MBC of the formulation were also performed as described.

## *Disc Agar Diffusion test (DAD*)

The disk diffusion method was also used to measure antimicrobial activity. For microbial strains, prepared new suspensions separately were inoculated with sterile swabs on BHI agar medium and cultured as grass. In the next step, each sample and formulation (the appropriate concentration of each extract in the formulation were obtained after the determination of the MIC and MBC of each sample) in the amount of 50 μl were poured on sterile blank paper discs with a diameter of 6 mm (Padtan Teb, Iran) to be absorbed by the disc. The discs were then placed on the culture medium with sterile forceps at a distance of 24 mm from each other. After placing the plates at the appropriate temperature and time, the diameter of the bacterial growth inhibition zone around the disk was measured in millimeters. Physiological serum was used for negative control. For positive control, 0.2% chlorhexidine gluconate mouthwash was used. The experiments were repeated three times.

### The biofilm formation evaluation

To perform this test, the micro-dilution method was used. Pathogenic bacteria were cultured for 18–24 h in TSB, complete with 1% turbidity sucrose equivalent to half McFarland. The suspension was diluted 1: 100 in TSB with 1% sucrose. Each extract with a twice concentration of MBC and the formulation separately was used to affect the rate of biofilm formation. 200 μl of an equal volume mixture of bacterial suspension and extract solution (100 μl suspension of pathogenic bacteria and 100 μl of extract and mouthwash) was transferred to each well in the microplates. Negative control wells contained 200 μl of the same volume mixture of bacterial suspension and physiological saline, and 0.2% chlorhexidine was used as a positive control. The plate surface was then covered and incubated in the incubator for 24 h at 37 °C. After 24 h, the contents of the well were removed, and the wells were washed three times with phosphate saline buffer. This was done due to the removal of disconnected cells. At this stage, the cells adhering to the bottom of the well were stained with 1% violet crystal and filled with 200 μl of 33% glycolic acetic acid. After 15 min, the optical density of each well at 570 nm was read by an ELISA reader, and the biofilm formation rate (%) was calculated using the following equation:

$$\mathrm{The\,biofilm\,formation\,rate}=\frac{\mathrm{Samples\,}(\mathrm{OD})}{\mathrm{Control\,}(\mathrm{OD}) }\times 100$$

where OD _treatment_ and OD _control_ refer to the absorbance at 570 nm in each well with and without the samples, respectively, after the addition of dissolving solution.

### The biofilm degradation evaluation

A biofilm degradation activity test was also performed to investigate the destructive activity on biofilm by the micro-dilution method. Biofilms were formed from TSB medium with synthetic saliva (McDougall solution), 3% glucose, and inoculation of bacteria and fungi in 96-well microplates. The resulting composition was incubated for 24 h at 37 °C. As soon as the biofilm was made, the rest of the culture medium was discarded. The extracts were obtained at twice the concentration of MBC, and the formulation solution was added and then incubated again for 24 h at 37 °C. The biofilms attached to the well's walls were washed using phosphate buffer. 1% violet crystal was added to the walls and placed there for 15 min. The wells were then rinsed with sterile water three times, and 95% alcohol was added. The suspension was incubated for 45 min, and the solution was transferred to a new microplate. The optical density of the suspension in each well was measured using a microplate reader with a wavelength of 570 nm to determine the extent of biofilm degradation. Chlorhexidine 0.2% was used for positive control and physiological serum for negative control. The biofilm reduction rate (%) was calculated using the following equation:

$$\mathrm{The\,biofilm\,reduction\,rate}=100-\left(\frac{\mathrm{Samples\,}(\mathrm{OD})}{\mathrm{Control\,}(\mathrm{OD}) }\right)\times 100$$

where OD treatment and OD control refer to the absorbance at 570 nm in each well with and without the samples, respectively, after the addition of dissolving solution.

### Statistical analysis

All tests were independently performed in triplicates. The results obtained were analyzed using a one-way ANOVA test accompanied by a Tukey post hoc test to compare means among groups. The importance level became set at *p* ≤ 0.05. Statistical evaluation was carried out with SPSS statistics model 20.

## Results

### Phytochemical analysis

The results of the determination of total phenolic and flavonoid compounds from the samples are illustrated in Table 1. *Arctium lappa* had the lowest total phenolic and flavonoid value of the samples. On the other hand, the total phenolic content of *Zataria multiflora* and the total flavonoid content of *Echinacea purpurea* were the highest.

**Table 1:** The total content of phenol and flavonoid contents

Samples	Total phenol (mg/g)	Total flavonoid (mg/g)
*Arctium lappa*	19.35	12.29
*Echinacea purpurea*	22.99	86.21
*Zateria multiflora*	151.69	77.23

**Fig. 2:**
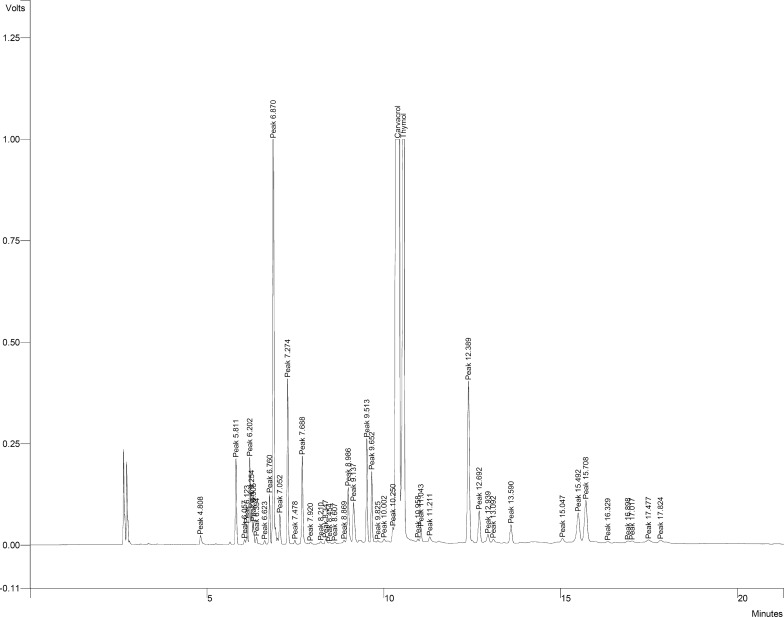
Gas chromatogram of *Zataria multiflora* oil extract showing thymol and carvacrol as the major constituents

Figure 2 illustrates the gas chromatogram of the GC-MS analysis of *Zataria multiflora* EO. Two major peaks were identified as thymol and carvacrol from the GC-MS database. Thymol and carvacrol content in *Z. multiflora* EO were measured at 310,740 and 424,000 ppm, equal to 31% and 42.2%, respectively.

### Antimicrobial analysis

The MIC and MBC values of each sample are presented in Table 2. In this study E, Z, A, and F stand for the samples of *Echinacea purpurea, Zataria multiflora, Arctium lappa,* and the formulation. *Echinacea purpurea* had a bacteriostatic and bactericidal effect against almost all tested samples and had lower concentrations than *Arctium lappa.*

**Table 2:** MIC, MBC, and MFC of *Echinacea, Arctium, and Zataria*

Bacteria	*Echinacea purpurea*		*Arctium lappa*		*Zataria multiflora*	
	MIC (mg/ml)	MBC/MFC (mg/ml)	MIC (mg/ml)	MBC/MFC (mg/ml)	MIC (%)	MBC/MFC (%)
*C. albicans*	375	750	750	> 750	0.187	0.375
*S. mitis*	187.5	187.5	187.5	375	0.023	0.046
*S. mutans*	93.75	187.5	187.5	375	0.011	0.011
*S. salivarius*	93.75	187.5	187.5	375	0.011	0.022
*L.acidophilus*	187.5	375	375	750	0.046	0.092
*E. coli*	375	750	375	750	0.023	0.046
*S. aureus*	750	> 750	750	> 750	0.046	0.092

All samples were tested three times in independent experiments

Taking the MBC values of the three herbal samples into consideration, the initial concentrations of herbal extracts in the formulation was suggested as follows: E: 187.5 mg/ml, A: 187.5 mg/ml, Z: 2.81 µl/ml. Table 3 illustrates MIC and MBC test results that showed the restricted growth of *S. mutans, S. salivarius, S. mitis, E.coli,* and *S. aureus* at the concentrations of 2.93 mg/ml for E and A and 0.04 µl/ml for Z which means the fraction 1/64 of primary concentration of A, E, and Z in the formulation could inhibit the growth of these bacteria. Moreover, the MBCs and MFCs were twice the value of MICs for all samples, which means a formulation consisted of *Zataria* essential oil at the lowest concentration of 0.08 µl/m, and Burdock and Echinacea extracts at the concentrations of 5.86 mg/ml could significantly reduce Streptococcus species associated with dental caries completely.

**Table 3:** MIC, MBC, and MFC of the formulation

Bacteria	Herbal Formulation	
	MIC (A,E: mg/ml- Z: µl/ml)	MBC/MFC (A,E: mg/ml-Z: µl/ml)
*C. albicans*	A: 5.86, E: 5.86, Z: 0.08	A: 11.72, E: 11.72, Z: 0.16
*S. mutans*	A: 2.93, E: 2.93, Z: 0.04	A: 5.86, E: 5.86, Z: 0.08
*S. mitis*	A: 2.93, E: 2.93, Z: 0.04	A: 5.86, E: 5.86, Z: 0.08
*S. salivarius*	A: 2.93, E: 2.93, Z: 0.04	A: 5.86, E: 5.86, Z: 0.08
*L. acidophilus*	A: 5.86, E: 5.86, Z: 0.08	A: 11.72- E: 11.72, Z: 016
*E. coli*	A: 2.93, E: 2.93, Z: 0.04	A: 5.86, E: 5.86, Z: 0.08
*S. aureus*	A: 2.93, E: 2.93, Z: 0.04	A: 5.86, E: 5.86, Z: 0.08

All samples were tested three times in independent experiments

A: *Arctium lappa*, E: *Echinacea purpurea*, Z: *Zateria multiflora*

In Table 4, the diameters of inhibition zones of samples are illustrated. The mean diameter of the inhibition zone for the formulation against S. mutans was 20 mm, which is the highest number and most effective compared to other species. However, the inhibition zone diameter was determined against *Candida albicans* (14 mm). Zones of Z and F inhibition against *S. mutans* had no significant difference with the gold standard, Chlorhexidine 0.2% (*p* > 0.05%).

**Table 4:** Mean value of zones of inhibition (mm) of A, E, Z, F, and CHX 0.2% in Disc Agar Diffusion test

Bacteria	MBC/MFC concentrations				CHX 0.2%
	F	Z	E	A	
*C. albicans*	14	13.66	11.66	10	**20**
*S. mutans*	20^*^	19.66^*^	18	13.33	**20.66**
*S. mitis*	16	14.66	16.66	13	**21.33**
*S. salivarius*	17.66	16.66	15.33	14	**21**
*L. acidophilus*	14.33	11.66	10.66	11.33	**21.66**
*E. coli*	16.33	15	14	12	**21**
*S. aureus*	15	14.33	11.66	10	**20**

All samples were tested three times in independent experiments

A: *Arctium lappa*, E: *Echinacea purpurea*, Z: *Zateria multiflora,* F: Formulation, CHX: Chlorhexidine

^*^shows the insignificant difference (p > *0.05) with Chlorhexidine 0.2%*

The effect of samples on the prevention of biofilm formation was tested using the micro-dilution method. Biofilm formation rates of samples are reported in percentage in Table 5. These percentages compare the biofilm formation of the tested microorganisms during exposure of different samples with the control group by measuring the optical density of each well at 570 nm. The formulation had a significant effect against the biofilm formation of all tested bacteria (*p* < 0.001) and *Candida albicans* (*p* = 0.02). However, chlorhexidine 0.2% as the positive control had a significantly higher effect than other samples (*p* < 0.001). *Arctium lappa* had no significant effect against all tested biofilms compared to the control group (*p* > 0.05). *Zataria multiflora* was the most effective sample against biofilm formation among Z, A, and E. On top of that, it must be stated that the formulation had a significantly higher anti-biofilm effect compared with Z.

**Table 5:** The biofilm formation percentages of species treated with samples of A, E, Z, F, and chlorhexidine 0.2%

Bacteria	OD (570 nm)				CHX 0.2%
	F%	Z%	E%	A%	
*C. albicans*	79^*^	80^*^	84	95	19^***^
*S. mutans*	48^***^	72^**^	79^**^	91	15^***^
*S. mitis*	57^***^	75^**^	82^*^	86	16^***^
*S. salivarius*	56^***^	70^**^	84	90	17^***^
*L. acidophilus*	69^***^	75^***^	82^**^	90	19^***^
*E. coli*	70^***^	70	84	90	17^***^
*S. aureus*	65^***^	75	82*	90	19^***^

All samples were tested three times in independent experiments

A: *Arctium lappa*, E: *Echinacea purpurea*, Z: *Zateria multiflora,* F: Formulation, CHX: Chlorhexidine

Significance level of *p* < 0.05 (*); or *p* < 0.01 (**); or *p* < 0.001 (***) in comparison with control group

The effect of extracts on biofilms was tested using the same method. In this case, the percentage biofilm reduction rate was calculated and illustrated in Table 6. The formulation had a significant effect on all groups of biofilms (*p* < 0.001). However, its effectiveness was significantly lower than Chlorhexidine 0.2% (*p* < 0.001). Sample E had a significant effect only against *L. acidophilus* biofilm (*p* = 0.015), while sample A effect on biofilm reduction was not significant (*p* > 0.05). The most biofilm reduction rate of samples F and Z was observed against *S. mitis* (35%, 17%, respectively).

**Table 6:** The biofilm reduction percentage of species treated with samples of A, E, Z, F, and chlorhexidine 0.2%

CHX 0.2%	OD (570 nm)				Bacteria
	F%	Z%	E%	A%	
73^***^	24^***^	12	8	2	*C. albicans*
75^***^	24^***^	9	3	3	*S. mutans*
74^***^	35^***^	17^**^	12	4	*S. mitis*
78^***^	23^**^	13	9	3	*S. salivarius*
72^***^	23^***^	15^**^	13^*^	5	*L. acidophilus*
78^***^	30^***^	12	9	2	*E. coli*
75^***^	25^***^	10	11	2	*S. aureus*

All samples were tested three times in independent experiments

A: *Arctium lappa*, E: *Echinacea purpurea*, Z: *Zateria multiflora,* F: Formulation, CHX: Chlorhexidine

Significance level of *p* < 0.05 (*); or *p* < 0.01 (**); or *p* < 0.001 (***) in comparison with control group

### Cell viability evaluation

**Fig. 3:**
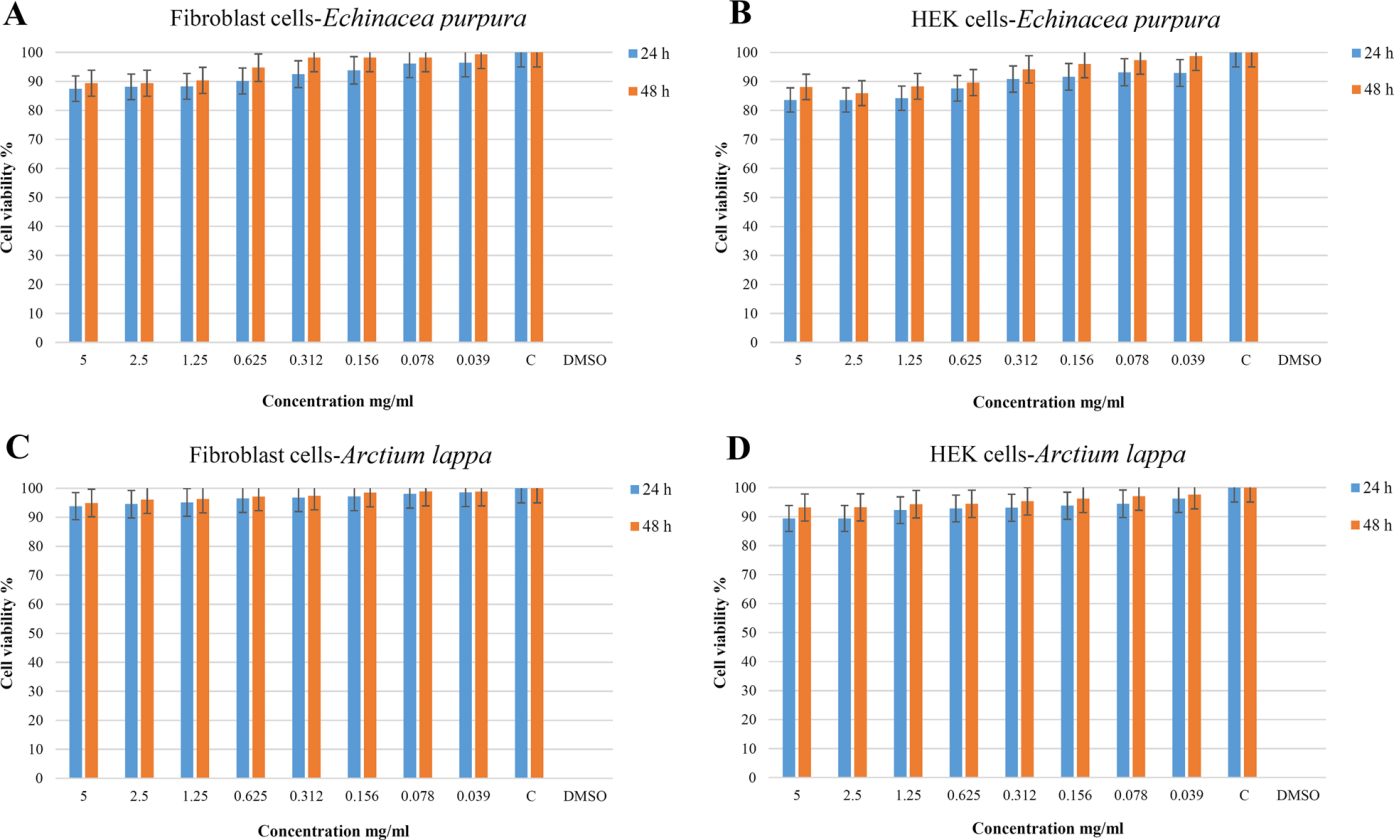
Cell viability after treatment with *Echinacea* and *Arctium* extracts and the formulation after 24 and 48 h. **A** The HDFa cells viability of *Echinacea purpurea*: There were no significant differences with the control group for 0.312 mg/ml concentrations and lower after 24 h (*p* > 0.05). This threshold for an incubation time of 48 h would be 0.625 mg/ml. **B** The HEK cells viability of *Echinacea purpurea*: There were no significant differences with the control group for the concentrations of 0.625 mg/ml and lower after 24 h (*p* > 0.05). This threshold for an incubation time of 48 h would be 0.156 mg/ml. **C** The HDFa cells viability of *Arctium lappa*: There were no significant differences with the control group for 1.25 mg/ml and lower after 24 h (*p* > 0.05). This threshold for an incubation time of 48 h would be 2.5 mg/ml. **D** The HEK cells viability of *Arctium lappa*: There were no significant differences with the control group for all tested concentrations after 24 or 48 h (*p* > 0.05)

**Fig. 4:**
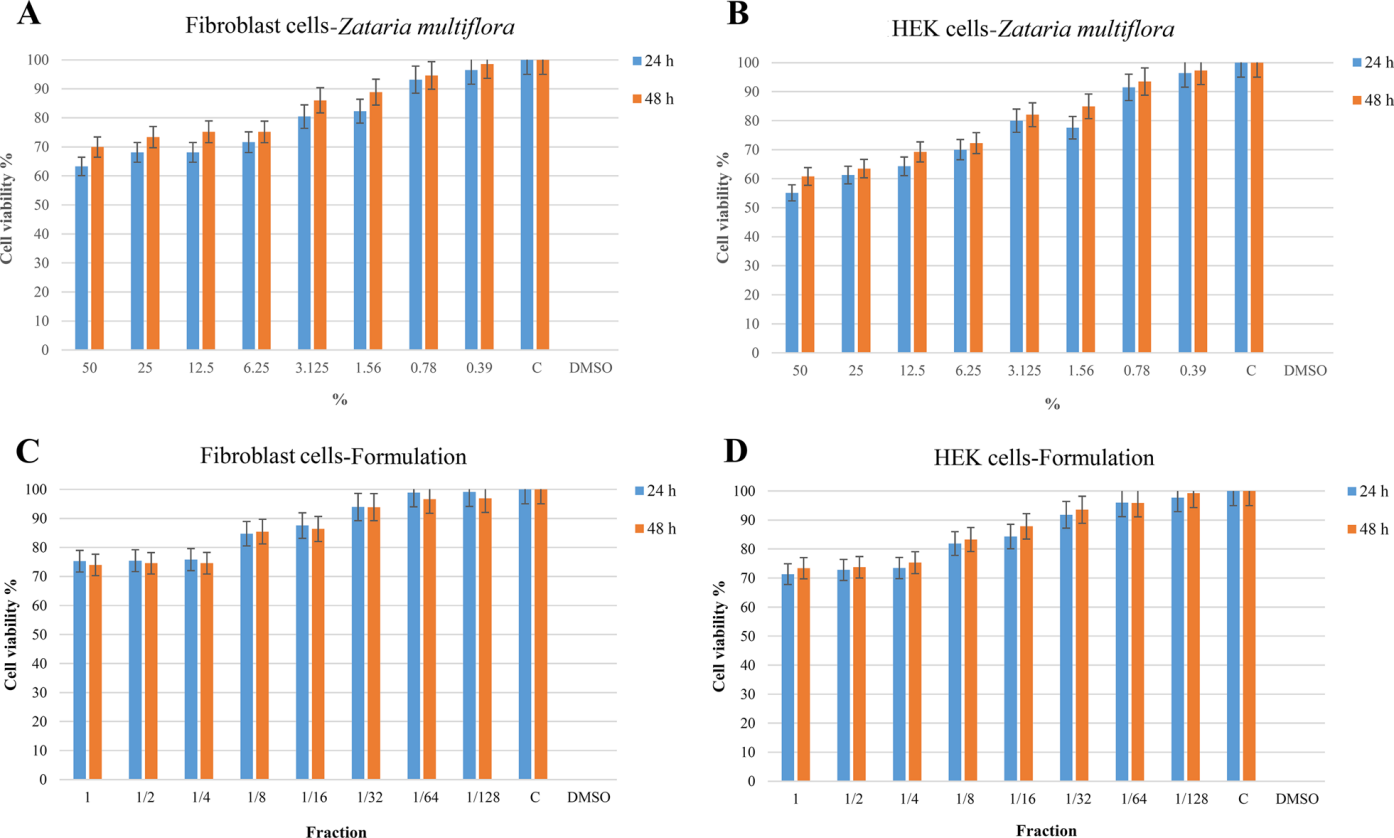
Cell viability after treatment with *Zataria* EO and the formulation after 24 and 48 h. **A** The HDFa cells viability of *Zataria multiflora*: There are no significant differences with the control group for 0.78% mg/ml concentration and lower after 24 h (*p* > 0.05). This threshold for an incubation time of 48 h would be 1.56%. **B** The HEK cells viability of *Zataria multiflora*: There are no significant differences with the control group for 0.78% mg/ml concentration and lower after 24 h or 48 h (*p* > 0.05). **C** The HDFa cells viability of the formulation and its serial dilutions: There was no significant difference with the control group for the volume concentration of 1/64 and lower after 24 h (*p* > 0.05). This threshold for an incubation time of 48 h would be 1/32. **D** The HEK cells viability of the formulation and its serial dilutions: there was no significant difference with the control group for the volume concentration of 1/32 and lower after 24 or 48 h (*p* > 0.05)

To determine the possible effect of E, A, Z and formulation on cell growth, the two types of cells (HEK 293 and HDFa cells) were incubated with E (39–5000 µg/mL), A (39–5000 µg/mL), Z (0.39%-50%) and F (1/128-1) for 24 and 48 h, and the cell viability was measured using MTT assay. A significant reduction of viable cells was observed using extracts in a dose and time-dependent pattern. By determining of optical density of vital cells after treatment with the samples for incubation times of 24 and 48 h, the cell viability percentage for both cell lines was calculated compared to the control group. These percentages are illustrated in Figs. 3 and 4.

It was observed that cell viability was decreased by increasing the concentrations, but these decreases were not significant below a specific concentration for each of these samples (*p* > 0.05). The cell viability percentages were higher than 50% in the tested concentrations for all groups.

#### Discussion

The establishment of pathogenic microorganisms can cause serious health issues, especially infections associated with biofilm. As a result, discovering anti-biofilm agents is essential and provides an effective tool for preventing oral cavity infections. The oral colonizer *S. mutans* is the most common. The bacterium adheres to the tooth surface, creating ideal conditions for secondary colonizers, including *Lactobacillus* species, to colonize. Plaque management is therefore crucial, with natural antimicrobial mouth rinses complementing and synergistically assisting mechanical plaque elimination. Herbal mouthwashes are moderately effective and less toxic than prescription mouthwashes like chlorhexidine (de Oliveira Carvalho et al. 2020). This research focuses on the anti-carious effect of *Zataria multiflora*, *Echinacea purpurea*, and *Arctium lappa* extracts on cariogenic bacteria and *Candida albicans*.

*Zataria multiflora* has long been used in traditional Iranian medicine. *Zataria multiflora* has antimicrobial, antioxidant, anti-inflammatory, antispasmodic, and analgesic effects as an edible plant and valuable medicine, according to clinical and pharmacological studies (Khazdair et al. 2018). This plant's essential oils contain a high percentage of oxygen monoterpenes, especially thymol and carvacrol, and have antimicrobial properties. In general, the antimicrobial property of this plant appears to be its most intriguing biological impact (Niczad et al. 2019). In *Zataria multiflora*, Saei Dehkordi discovered that a high percentage of thymol and carvacrol induced antiseptic properties. Because of their lipophilic origin and attachment to the cell membrane of bacteria, thymol and carvacrol disrupt bacteria's activity. The essential oil can withstand high temperatures and a wide variety of pH levels (Saei-Dehkordi et al. 2010).

Alipour et al. (2018) developed and tested an herbal mouthwash containing oak bark extract and *Zataria* essential oils. The researchers studied formulations containing 0.15% *Zataria* essential oil obtained by hydro-distillation and various amounts of tannin, the active ingredient in oak bark extract. In this analysis, the best formulation had tannin concentrations of 0.2 and 0.5 percent and significantly outperformed Persica mouthwash in terms of antimicrobial effect. The active ingredients thymol and carvacrol in *Zataria multiflora* were found to have 44 and 23 percent, respectively (Alipour et al. 2018). In 2010, Owlia et al. (Owlia et al. 2010) conducted research on the antimicrobial effects of *Zataria multiflora* on caries-causing streptococci in teeth. The MICs for *S. sanguis, S. salivaius,* and *S. mutans* were 15%, 20%, and 10%, respectively. These bacteria had MBC values of 25%, 20%, and greater than 25%, respectively. The number of bacteria that died was influenced by the amount of the extract and the duration of exposure. In this way, as the concentration and exposure time of the extract improved, so did its effectiveness against bacteria. Finally, the writers recommend using this plant as a medication (mouthwash), which was done in this article. Sharififar et al. (2007) found that *Zataria multiflora* has a more significant effect on gram-negative bacteria (Sharififar et al. 2007), while Dehkordi et al. 2010 compared Shirazi thyme from different Iranian regions and discovered that gram-negative bacteria are more resistant to it. *Escherichia coli* O157:H7 was the most resistant organism, and *Candida tropicalis* was the most susceptible, with MICs of 16 and 0.062 mg/ml, respectively (Saei-Dehkordi et al. 2010). *Zataria multiflora* essential oil has been involved in eliminating *Enterococcus faecalis* and cleaning the dental canal (Ravanshad et al. 2007). Khakzad et al. investigated the cytotoxic effects of *Satureja khuzistanica* and *Zataria multiflora* essential oils on the growth inhibition of PBMC and K562 cell lines. Hydro-distillation was used to collect the essential oils from the plants. The MTT test was used to assess the effects of four different concentrations (12.5, 25, 50, and 100 g/ml) of *Z. multiflora* essential oils on cell viability in PBMC and K562 cell lines after 24, 48, and 72 h of incubation. For the standard PBMC and K562 cell lines, the 50% growth inhibitory concentration (IC50) of *Z.multiflora* oil was 64.97 and 42.82 (µg/mL), respectively (Khakzad et al. 2019).

Few studies have investigated *Echinacea*'s antibacterial activity in dental caries. Still, its antibacterial effect on respiratory bacteria such as *Hemophilus influenzae* and *Legionella pneumophila* is due to its dual anti-inflammatory and bactericidal effects (Sharma et al. 2010). High anti-inflammatory properties, helping to heal mucosal wounds, and strong anti-viral effect made it very beneficial to be included in this formulation as an adjuvant extract. *Echinacea* has been found to affect gingivitis severity and duration and dental plaque. Owing to the benefits of herbal compounds over chemical mouthwashes, like fewer side effects, a comparison of Echinacea solution and chlorhexidine on dental plaque and gingivitis improvement reveals that Echinacea extract solution has significantly improved decreased dental plaque and the incidence of gingivitis as compared to chlorhexidine. Hence, it can be considered a suitable alternative to chlorhexidine (Safarabadi et al. 2017). Fathi et al. discovered that *S.officinalis, L.citriodora, and M.piperita* essential oils, but not *E.purpurea* and *M.chamomilla* extracts, could dissolve the biofilms formed by the oral pathogen bacteria. The herbal mixture of these extracts decomposed the biofilm on the teeth in 45 s. According to light and electron microscopy findings, the bacterial structure exposed to the herbal mixture was deformed (Fathi et al. 2021). Izadi et al. 2020 demonstrated that the ethanolic extract contained the highest total phenolic content and cichoric acid. The antimicrobial ability of purple coneflower aqueous and ethanolic extracts were higher in the ethanolic extract with a 3000 g/ml concentration. At doses of 25 and 50 mg/ml, the aqueous extract of purple coneflower has no effect on gram-negative bacteria growth. According to the findings, the ethanolic extract had a larger inhibitory effect on the examined strains than the aqueous extract. Depending on the bacteria, the MIC of purple coneflower ethanolic extract ranged from 16 to 256 mg/ml (gram-positive or gram-negative) (Izadi et al. 2020). Wang et al. assessed the cytotoxic effect of Echinacea extracts prepared from the whole plant, stem and leaf, flower, and root to analyze the viability of human dendritic cells (DCs). Except for the whole plant group, none of the extracts were cytotoxic at 1 to 100 g/ml (Wang et al. 2006).

The lyophilized extract of burdock leaves has been shown to have antimicrobial activity against oral microorganisms, mainly bacteria linked to endodontic pathogens such as *Bacillus subtilis, Lactobacillus acidophilus, and Pseudomonas aeruginosa* (Pereira et al. 2005). The MIC and MBC concentrations of the extract of A. lappa on *B. subtilis* were 600 and 750 mg/ml, respectively. Also, these values were 230 and 540 mg/ml for *H. influenza* (Habibipour et al. 2015). Chlorogenic acid isolated from the extract of this plant has shown inhibitory effects on *Escherichia coli, Micrococcus luteus,* and *Staphylococcus aureus* (Xuezheng et al. 2004). Oliviera et al. evaluated the antimicrobial activity of *Arctium lappa L.* extract on *Staphylococcus aureus, S. epidermidis, Streptococcus mutans, Candida albicans, C. tropicalis,* and *C. glabrata*. In addition, the cytotoxicity of this extract was analyzed on macrophages (RAW 264.7).The most effective concentration was 250 mg/mL and also promoted significant reduction in the biofilms of *S. aureus, S. epidermidis, S. mutans* and *C. albicans*. Cell viability was similar to 100% (de Oliveira et al. 2014).

In the present study, the formation and degradation of biofilm showed that although *Zataria multiflora* essential oil (twice the concentration of MBC) was effective on the biofilm formation and degradation of bacterial and Candida species, this effect was more significant when combining *Zataria multiflora* essential oil with Burdock and *Echinacea* extracts. However, the extract of Burdock and *Echinacea* alone had a limited effect on the biofilm growth of the tested species of the bacteria and *Candida albicans*. These findings revealed that the formulation containing the essential oil of *Zataria multiflora* and extracts of *Echinacea* and Burdock inhibited the development of *Streptococcus mutans* biofilm by more than half and had the most significant effects on *Streptococcus mutans* as compared to other bacterial strains and Candida. In terms of biofilm degradation, it had the most considerable effect on *Streptococcus mitis*.

As to what limitations the study has, it is in-vitro research, and the formulation needs to be tested clinically in future research. The other limitation is that different extraction methods obtained from different parts of plants from different locations could compare to obtain the optimum thymol level, carvacrol, total flavonoid, and phenolic content. Nonetheless, the amount of these components in the extracts was at acceptable levels. While having a noticeable impact on bacterial organisms, the formulation was less effective than chlorhexidine 0.2% mouthwash. However, since chlorhexidine mouthwash has many side effects when used for a long time, including tooth pigmentation, changes in taste, burning and dry mouth, flatulence of the gums, and side effects from potential swallowing (Jones 1997), using herbal mouthwash without any significant side effects can be a beneficial alternative, even with a lower antimicrobial and biofilm impact. This mouthwash is purely herbal without adding alcohol or any other additives as other products found on the market. Antimicrobial studies specifically demonstrate that this formulation is a potent plaque inhibitor that could be used as a promising ingredient in herbal mouthwash. The mixture blocked plaque formation and degraded biofilm formation and had no cytotoxic effects on normal human cells at the concentrations used. However, further long-term clinical trials are required to include the much-needed standardization and certification of the mouthwash to overcome the drawbacks of the standard gold chlorhexidine.

## Data Availability

All the data generated or analyzed during this study are included in this published article, and also, the datasets analyzed to support the findings of this study are available from the corresponding author upon request.
